# Mucosal incision assisted unroofing technique for endoscopic full thickness resection of a gastric subepithelial lesion

**DOI:** 10.1055/a-2717-1635

**Published:** 2025-10-29

**Authors:** Taylor Bowler, Rahul Karna, Timothy Davie, Carlos Iwamoto, Mohammad Bilal

**Affiliations:** 1 5635 Division of Internal Medicine, University of Minnesota Medical Center, Minneapolis, Minnesota, United States; 2 5635 Division of Gastroenterology and Hepatology, University of Minnesota Medical Center, Minneapolis, Minnesota, United States; 3 20040 Department of Laboratory Medicine and Pathology, Veteran Affairs Medical Center, Minneapolis, Minnesota, United States; 4 12225 Division of Gastroenterology and Hepatology, University of Colorado Anschutz Medical Campus, Aurora, Colorado, United States

Endoscopic full thickness resection (EFTR) using a full thickness resection device (FTRD; OVESCO Endoscopy AG, Tuebingen, Germany) is a safe and effective technique for the management of subepithelial lesions (SELs; 1 ). This technique utilizes a grasping forceps to facilitate retraction into the FTRD prior to clip deployment followed by resection using snare with electrocautery. However, this technique can be challenging with SELs since the grasping forceps often capture the mucosa leading to slippage of the underlying SEL. This limitation can be overcome with mucosal incision and unroofing to expose the underlying lesion and allow for EFTR using the FTRD. This video case report outlines the technique of EFTR using the FTRD after mucosal unroofing of a gastric SEL ( Video 1 ).

Video image: the subepithelial lesion after the successful unroofing technique.Video 1

A 65-year-old male with history of tobacco use was referred for the management of a gastric SEL. Endoscopic ultrasound demonstrated a 11 mm × 5 mm hypoechoic lesion originating from the submucosa (layer III) of the gastric body. Prior fine needle biopsies were non-diagnostic. Endoscopic examination with white light imaging showed a 11 mm subepithelial lesion with a negative pillow sign ( Fig. 1 **a** ). As demonstrated in the video, initial mucosal incision was performed with an endoscopic submucosal dissection knife to unroof the underlying lesion ( Fig. 1 **b** ). Exposure of the lesion beneath the mucosal surface allowed for direct grasping of the lesion with the grasping forceps, retraction into the FTRD cap, and subsequent EFTR using the FTRD. EFTR was demonstrated by the fatty patch within the clip and the specimen was successfully retrieved ( Fig. 1 **c** ). Lesion histopathology showed pancreatic heterotopia involving the submucosa with focal mucosal erosion ( Fig. 1 **d** ). There were no adverse events with the procedure.

**Fig. 1:**
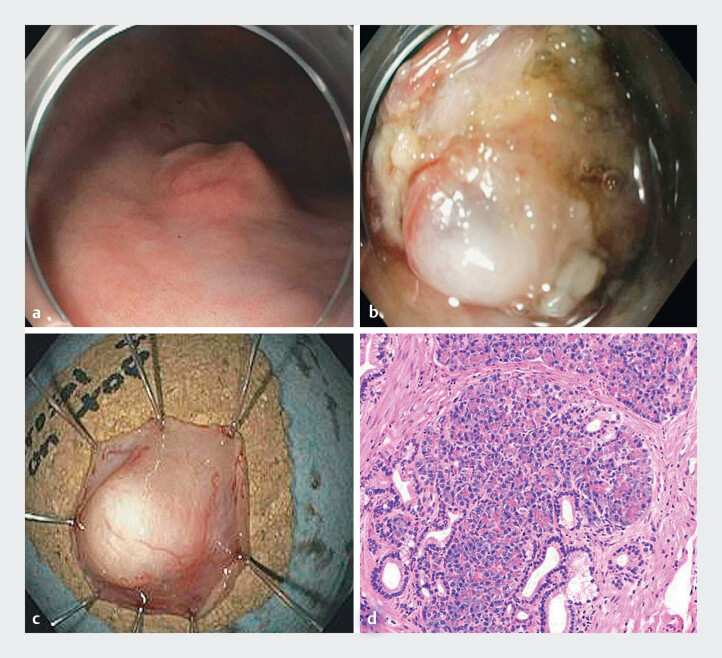
**a** SELs in the gastric body. **b** SELs after the mucosal unroofing technique. **c** Successful retrieval of the lesion after EFTR. **d** Lesion histopathology demonstrating pancreatic heterotropia.

This case highlights that mucosal unroofing can allow for successful EFTR for the resection of SELs. Future studies are needed to explore the utility of this technique compared to traditional endoscopic resection methods for the management of gastric SELs.

Endoscopy_UCTN_Code_TTT_1AO_2AG_3AD

## Notes

Endoscopy E-Videos https://eref.thieme.de/e-videos
*E-Videos* is an open access online section of the journal *Endoscopy* , reporting on interesting cases and new techniques in gastroenterological endoscopy. All papers include a high-quality video and are published with a Creative Commons CC-BY license. Endoscopy E-Videos qualify for HINARI discounts and waivers and eligibility is automatically checked during the submission process. We grant 100% waivers to articles whose corresponding authors are based in Group A countries and 50% waivers to those who are based in Group B countries as classified by Research4Life (see: https://www.research4life.org/access/eligibility/ ). This section has its own submission website at https://mc.manuscriptcentral.com/e-videos .
